# Therapeutic potential of stem cell-derived extracellular vesicles in aging and regeneration

**DOI:** 10.3389/fragi.2026.1752530

**Published:** 2026-04-30

**Authors:** Isobel K. Dunstan, Daniel C. Anthony, Francesca Lugarini, Sherif Idriss

**Affiliations:** 1 Department of Pharmacology, University of Oxford, Oxford, United Kingdom; 2 Lübeck Biosciences, Lübeck, Germany

**Keywords:** aging, exosomes, extracellular vesicles, immunomodulation, longevity, regenerative medicine, stem cells

## Abstract

Aging is characterized by measurable reductions in tissue repair, immune balance, and metabolic regulation. Increasing evidence suggests that these changes may arise, in part, from an insufficiency or altered quality of endogenous extracellular vesicle (EV) signaling. EVs, including exosomes, carry regenerative and immunoregulatory cues, and age-related alterations in their abundance, cargo, and bioactivity have been linked to impaired cellular communication across organ systems. This has fueled growing interest in stem cell-derived EVs, which provide biologically more youthful vesicles that reproduce key paracrine functions of their parent cells while avoiding the limitations of cell transplantation. By transferring defined protein, lipid, and RNA cargoes, these vesicles influence pathways central to aging biology, including mitochondrial function, inflammatory control, and maintenance of stem cell niches. Preclinical studies support their efficacy in models of neurodegeneration, wound healing, musculoskeletal decline, and systemic inflammation. However, their function depends on stem cell origin, donor age, and environmental conditioning, variables that complicate standardization and clinical scalability. As interest expands across therapeutic and cosmetic domains, a comparative understanding of EV sources and their mechanistic actions is required. In this review, we examine stem cell-derived EVs across biological sources, outline how aging and environmental factors shape their regenerative potency, and evaluate current progress in clinical translation. The field has reached a point where future advances depend less on further demonstrations of efficacy and more on resolving challenges related to manufacturing, quality control, and regulatory alignment. Addressing these constraints will determine whether stem cell-derived EVs can progress from experimental promise to practical interventions for aging and regenerative medicine.

## 1 Introduction

Extracellular vesicles (EVs) are nanoscale, lipid bilayer-enclosed particles secreted by virtually all cell types, carrying proteins, lipids, nucleic acids, and metabolites that enable intercellular and inter-organ communication (Doyle and Wang, 2019). EVs are often categorized into three broad categories according to their biogenesis: exosomes (30–150 nm), which originate from the endosomal system via multivesicular body fusion with the plasma membrane; microvesicles (100–1,000 nm), which bud directly from the plasma membrane; and apoptotic bodies (50–5,000 nm), which are released during programmed cell death and often contain nuclear fragments and organelles (Doyle and Wang, 2019; Yanez-Mo et al., 2015). In practice, however, these categories overlap substantially in size and molecular composition, and current isolation techniques do not reliably distinguish between them (Welsh et al., 2024). For this reason, and in keeping with the recommendations of the International Society for Extracellular Vesicles (ISEV), we use the inclusive term extracellular vesicles (EVs) to encompass vesicles of endosomal or plasma membrane origin without presuming a specific biogenetic pathway (Welsh et al., 2024).

EVs have been implicated in diverse physiological and pathological processes, including immune modulation, angiogenesis, neurovascular support, tissue repair, and cancer progression (De Jong et al., 2014; Chen et al., 2024). They influence recipient cells by transferring proteins, lipids, RNAs, mitochondrial components, and metabolites, thereby reprogramming intracellular signaling and altering cellular phenotypes (Doyle and Wang, 2019). Within the context of aging, EVs contribute to several processes relevant to the hallmarks of aging. For example, they propagate chronic low-grade inflammation (“inflammaging”), carry and transmit signals that reinforce cellular senescence, and can impair or restore intercellular communication depending on their origin (Wang et al., 2018; Takasugi et al., 2017). Emerging evidence also suggests EVs influence mitochondrial function and bioenergetics by transferring mitochondrial proteins or RNAs, thereby intersecting with another key hallmark of aging (Chen et al., 2024; Phinney et al., 2015). This dual capacity to accelerate degenerative changes or deliver reparative cues underscores their centrality in age-related biology.

For therapy, among the most promising are stem cell-derived EVs (SC-EVs), which recapitulate many of the paracrine and immunomodulatory functions of their parent stem cells, such as promoting angiogenesis, enhancing tissue repair, and dampening pathological immune responses (De Jong et al., 2014; Chen et al., 2024). Unlike cell therapies, however, SC-EVs are not associated with risks of engraftment failure, alloimmune rejection, or teratoma formation (Rani et al., 2015; Matt et al., 2008). Their scalability, stability in storage, and ability to cross biological barriers, including the blood–brain barrier and the epidermis, provide additional advantages for clinical translation (Wiklander et al., 2019; Mentkowski et al., 2018; Kink et al., 2024; Trenkenschuh et al., 2022). Nevertheless, translation faces significant obstacles. EV cargo composition is shaped by the stem cell source (embryonic, mesenchymal, induced pluripotent, or tissue-specific), donor characteristics such as age and metabolic state, and environmental factors including hypoxia, inflammatory stimuli, and nutrient availability (Taylor and Shah, 2015; Mushahary et al., 2018; Alibhai et al., 2020; Abbasi Sourki et al., 2023; Nguyen et al., 2020). Isolation and characterization methods remain heterogeneous, complicating reproducibility and comparability across studies (An et al., 2018). The mechanisms by which specific EV cargoes mediate functional effects are incompletely defined. However, a central premise of EV-mediated communication is that cargo incorporation is not random, but selectively regulated, enabling cells to transmit defined biological signals. Emerging evidence indicates that cargo sorting involves coordinated pathways, including ESCRT-associated machinery, lipid-dependent membrane organization, and RNA-binding protein–directed loading (reviewed comprehensively elsewhere (Dixson et al., 2023)). These processes are likely to contribute to the functional heterogeneity observed between EV preparations and represent a key barrier to standardization (Nguyen et al., 2020).

**FIGURE 1:**
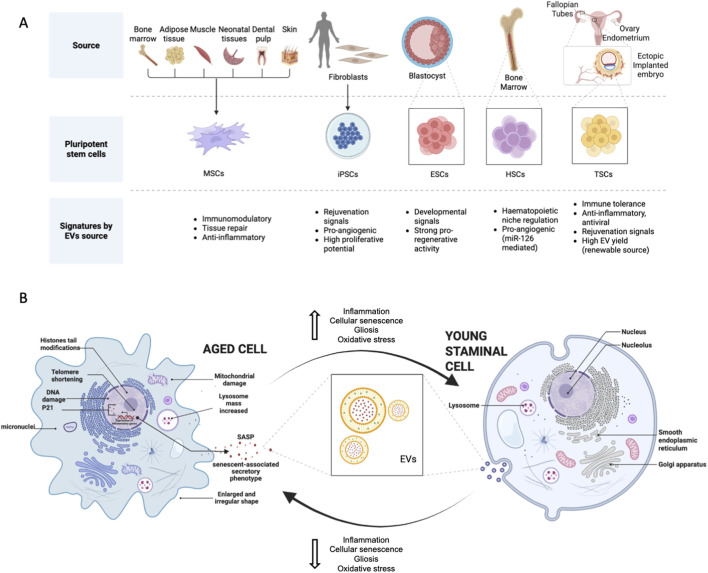
**(A)** Stem cell sources, EV functional signatures. The panel illustrates the major stem-cell sources used for EV production, including adult mesenchymal sources (bone marrow, adipose tissue, muscle, neonatal tissues, dental pulp, skin), induced pluripotent stem cells (iPSCs), embryonic stem cells (ESCs), hematopoietic stem cells (HSCs), and trophoblast stem cells (TSCs). Key functional signatures associated with EVs from each source are summarized, highlighting immunomodulatory, regenerative, angiogenic, developmental, and anti-inflammatory properties. **(B)** Bidirectional age-dependent effects of extracellular vesicles on cellular aging and rejuvenation. Aged EVs typically impair repair processes, heighten inflammatory and senescence-associated signalling, worsen metabolic and mitochondrial stress and reduce regenerative capacity. In contrast, young EVs can deliver pro-reparative, anti-inflammatory, and anti-senescence cargo that restores metabolic and mitochondrial function, reduces SASP factors, and enhances tissue repair, angiogenesis, neuroprotection, and stem-cell function.

Against this backdrop, stem cell-derived EVs are emerging as both powerful research tools and promising therapeutic candidates for aging and regenerative medicine. By situating their activity within established hallmarks of aging - particularly inflammation, senescence, intercellular communication, and mitochondrial dysfunction - we can begin to delineate where EV-based therapies are most likely to deliver clinical impact. A rigorous synthesis of their biological actions, determinants of variability, and translational barriers is required to move beyond proof-of-concept and towards reproducible clinical application (Figure 1).

## 2 Sources of stem cell-derived extracellular vesicles

Stem cell-derived EVs (SC-EVs) inherit many of the physiological and functional features of their parent cells, but their molecular composition is dynamic rather than fixed. EV cargo (including microRNAs, proteins, lipids, and metabolites) reflects not only intrinsic cell identity but also extrinsic factors such as donor age, replicative history, oxidative stress, hypoxia, nutrient status, and inflammatory exposure (Kosuthova et al., 2024; Fichtel et al., 2022; Zhuang et al., 2022; Haraszti et al., 2019). This plasticity is further shaped by reciprocal EV exchange within the stem cell niche: immune cells, fibroblasts, endothelial cells, and stromal components release vesicles that can remodel stem cell behavior, thereby indirectly influencing the profile of SC-EVs themselves (Wu et al., 2024; Liu A. et al., 2020; Burgy et al., 2024). In this sense, SC-EVs represent an “integrated signal” of both stem cell programming and environmental conditioning. The choice of stem cell source is therefore critical, since each lineage imparts a distinct functional repertoire to its EVs (Table 1). Mesenchymal stem cell (MSC)-derived EVs, for example, are strongly associated with immunomodulation and tissue repair (Phinney et al., 2015; Lai et al., 2010; Morrison et al., 2017); induced pluripotent stem cell (iPSC)-derived EVs capture aspects of pluripotency but are also subject to variability in reprogramming fidelity (Adamiak et al., 2018; Palama et al., 2025); hematopoietic stem cell (HSC)-derived EVs influence immune reconstitution and hematopoietic niche regulation; embryonic stem cell (ESC)-derived EVs carry developmental signals with high regenerative potential but raise ethical and safety considerations (Chen et al., 2019; Khan et al., 2015); and trophoblast stem cell (TSC)-derived EVs, a more recent focus, show unique immune-modulatory and pro-angiogenic activities with potential relevance to neuroprotection and pregnancy-associated conditions (Okae et al., 2018; Go et al., 2023).

**TABLE 1:** Comparative properties of major stem cell sources used for EV production. The table contrasts mesenchymal stem cells (MSCs), induced pluripotent stem cells (iPSCs), trophoblast stem cells (TSCs), hematopoietic stem cells (HSCs), and embryonic stem cells (ESCs) across key biological features relevant to EV manufacturing, including differentiation potential, tumorigenicity, immunogenicity, telomere length, and *in-vitro* aging. Advantages and limitations for each source are summarized, highlighting differences in regenerative potential, scalability, variability, and ethical or safety considerations.

Feature	MSCs	iPSCs	TSCs	HSCs	ESCs
Source	Bone marrow, adipose tissue, umbilical cord	Reprogrammed somatic cells	Placenta or trophoblast layer of blastocyst	Bone marrow, peripheral blood, umbilical cord blood	Inner mass of blastocysts
Pluripotency	Multipotent	Pluripotent	Pluripotent (first trimester)/multipotent (term placenta)	Multipotent	Pluripotent
Differentiation potential	Limited to mesodermal lineages (bone, cartilage, fat)	Can differentiate into all cell types	Can differentiate into multi-lineage cells depending on source	Restricted to hematopoietic and immune lineages	All lineages
Ethical issues	Minimal	Minimal	Minimal	Minimal	Significant
Tumorigenic potential	Low	High	Low	Low	High
Immunogenicity	Low to moderate	Variable	Very low	Moderate	High
Telomere length	Short	Long	Comparable to ESCs	Short	Long
Oxidative stress & epigenetic memory	High	Variable	Low	High	Low
Rate of *in-vitro* aging	Fast	Slow	Slow	Fast	Slow
Advantages for EV use	• Abundant • Immuno-modulatory • Well studied	• Unlimited expansion • Avoids replicative senescence issues of other adult cells • Potent regenerative EV profile compared to MSC-EVs	• Immune-privileged • Anti-viral signatures • Stable expansion	• Immune-regulatory EV signaling; relevant to inflammation	• Very high proliferative capacity • Stable telomeres • Strong regenerative EV cargo
Limitations/Risks	• High donor-to-donor variability • Replicative senescence • Heterogeneous EV cargo • Poor scalability	• Reprogramming variability • Genomic instability • Manufacturing complexity	• Field is early; limited *in vivo* evidence published • Properties vary according to stage of placental development	• Restricted lineage potential • Poor scalability • Variable purity	• Ethical constraints • Genetic instability during expansion • Tumorigenicity

In the following sections, we examine each of these stem cell sources in detail, highlighting not only their shared features but also the distinctive molecular cargo and mechanistic pathways conveyed by their EVs. By framing SC-EVs in terms of both their lineage of origin and their responsiveness to environmental conditioning, we can begin to understand how source selection shapes translational potential across aging and regenerative medicine.

### 2.1 Mesenchymal stem cells (MSCs)

Mesenchymal stem cells (MSCs), expressing the canonical marker trio CD73, CD90, and CD105 are the most extensively studied source of therapeutic EVs (Lelek and Zuba-Surma, 2020). They are multipotent stromal cells capable of self-renewal and differentiation into mesenchymal lineages including osteoblasts, chondrocytes, and adipocytes (Pittenger et al., 1999). However, these markers are not unique to MSCs and do not define a single, uniform cell population. Instead, MSC phenotype and functional potential vary with tissue of origin, donor characteristics and culture conditions, meaning that the conventional marker profile is useful but not sufficient to capture biological equivalence across preparations. MSC characterization therefore requires more rigorous standardization for clinical applications (Mushahary et al., 2018; Phinney, 2012). MSCs can be isolated from diverse tissues such as bone marrow, adipose tissue, umbilical cord, placenta, periosteum, and synovium, making them broadly accessible and versatile, although almost all large-scale MSC production facilities now use perinatal tissues or adipose tissue rather than bone marrow aspirates (Pittenger et al., 1999; Zuk et al., 2001; Erices et al., 2000; Fukuchi et al., 2004; Sakaguchi et al., 2005; De Bari et al., 2001). Despite recognized challenges in their isolation, including variable yields, susceptibility to contamination by phenotypically similar stromal populations, and a lack of standardized protocols for isolation and expansion, MSCs remain attractive candidates for regenerative therapies owing to their broadly immunomodulatory properties (Phinney, 2012; Chan et al., 2006; Musial-Wysocka et al., 2019). However, poor engraftment and limited long-term survival of transplanted MSCs have shifted attention toward paracrine signaling as their principal mode of action, mediated largely by secreted EVs (Musial-Wysocka et al., 2019; Zhang et al., 2019; von Bahr et al., 2012; Arslan et al., 2013; Gnecchi et al., 2005). MSC-derived EVs are enriched in microRNAs, proteins and lipids that modulate inflammation, repair and recipient-cell behavior (Kou et al., 2022). These vesicles have demonstrated therapeutic potential across diverse preclinical models of disease, including cardiovascular disease, neurodegeneration, wound healing, and osteoarthritis (Lelek and Zuba-Surma, 2020; Bailey et al., 2022; Chen et al., 2023; Losurdo et al., 2020; Mei et al., 2025).

Two early studies helped establish causality (Lai et al., 2010; Bruno et al., 2009). In a mouse model of myocardial ischemia–reperfusion, Lai et al. isolated a 50–100 nm, CD9/Alix-positive (exosome markers) fraction by size-exclusion HPLC from human ESC-derived MSC conditioned medium; a single intravenous dose given 5 min before reperfusion reduced infarct size to a degree comparable to the unfractionated conditioned medium, indicating that the isolated EV fraction was sufficient to reproduce much of the cardioprotective activity of the total conditioned medium (Lai et al., 2010). Likewise, in glycerol-induced acute kidney injury in SCID mice, Bruno et al. showed that a single intravenous dose of differentially ultracentrifuged MSC microvesicles derived from human bone marrow significantly improved renal function, as measured by creatinine and blood urea nitrogen, and morphology and tubular-cell proliferation relative to saline control. The MSC-EVs produced a similar effect size in all measures to their cell counterparts, indicating that the therapeutic effect of MSC delivery was captured by paracrine signaling. These changes were lost when vesicles were trypsinized or when CD44/β1-integrin interactions were pharmacologically blocked, indicating receptor-mediated, uptake-dependent activity and were abrogated by RNase treatment, consistent with RNA-dependent cargo effects (Bruno et al., 2009). Notably, both studies used human-derived EVs administered in murine models, reflecting a common preclinical strategy but one that introduces cross-species considerations when interpreting mechanism and translational relevance. These studies also argue for exosome-vs. microvesicle-specific effects, but pre-date MISEV2018/2023 and would now be framed as MSC-EVs defined by separation parameters rather than by biogenesis labels, unless specific endosomal origin or plasma-membrane budding is demonstrated (Welsh et al., 2024). MISEV (Minimal Information for Studies of Extracellular Vesicles) guidelines, developed by the International Society for Extracellular Vesicles, provide consensus standards for EV nomenclature, isolation, and characterization to improve rigor and reproducibility across the field (Welsh et al., 2024).

Despite these promising results, several challenges for clinical translation remain. MSCs are heterogeneous, with proliferative capacity, differentiation potential and secretory profiles varying by tissue source, donor and culture conditions (von Bahr et al., 2012). This extends to EVs: a label-free LC–MS/MS comparison of exosomes from bone-marrow, adipose and umbilical-cord MSCs reported source-specific proteomic signatures, particularly among membrane proteins and pathway annotations (Wang et al., 2020a). Whether this translates into functional consequences was not tested. In fact, other findings demonstrate EVs from Wharton’s jelly and bone-marrow MSCs produced broadly comparable benefits in a neonatal hyperoxia model of bronchopulmonary dysplasia (improved alveolarization, reduced fibrosis, preserved peripheral microvasculature, ameliorated pulmonary hypertension and better lung function) (Willis et al., 2018). However, in this case, EV cargo was not profiled. Inconsistencies, such as in EV isolation workflows and outcome readouts between studies (for example, functional testing without composition analysis and vice versa) limit cross-study comparability. Head-to-head experiments that standardize EV production, characterization, dosing metrics and shared efficacy endpoints are therefore, needed to link cargo to function robustly, and to fully understand the nature and effect of EV variability.

A further complication, which will be discussed in more depth later, is replicative senescence of MSCs during *in vitro* expansion, which alters the MSC secretome and diminishes EV potency (Wagner et al., 2008; Voynova et al., 2022). This variability constrains scalability but also ties MSC-EV biology to hallmarks of aging, including cellular senescence and impaired intercellular communication. Addressing donor/source selection, culture conditions and senescence control, alongside development of robust potency assays and release criteria, will be essential for clinical translation of MSC-derived EVs (Adlerz et al., 2020).

### 2.2 Induced pluripotent stem cells (iPSCs)

Induced pluripotent stem cells (iPSCs), typically generated from adult somatic cells, such as dermal fibroblasts through molecular reprogramming, are an increasingly investigated source of therapeutic EVs, proposed to circumvent limitations of adult stem cells such as restricted lineage potential and replicative senescence (Prigione et al., 2010; Lapasset et al., 2011). Reprogramming somatic cells to pluripotency restores self-renewal and trilineage differentiation capacity, enabling renewable, patient-matched production (Shafa et al., 2018). In several models, iPSC-EVs have matched or exceeded the efficacy of iPSC transplantation while avoiding risks intrinsic to cell grafts, including genomic instability and teratoma formation (Adamiak et al., 2018; Lu et al., 2019). For example, in a mouse model of reperfused myocardial infarction, intramyocardial iPSC-EVs delivered 48 h post-injury produced greater day-35 recovery than iPSC grafts, as measured by improved left ventricular (LV) function/perfusion, lower LV mass and apoptosis. Notably, iPSC grafts formed teratomas whereas iPSC-EVs were non-tumorigenic, underscoring an efficacy–safety advantage of the acellular product (Adamiak et al., 2018).

However, variability introduced by reprogramming may complicate translation. Early-passage iPSC lines retain cell-of-origin DNA-methylation and transcriptional “memory,” biasing lineage choice (Lister et al., 2011; Kim et al., 2010; Polo et al., 2010; Ohi et al., 2011). For example, β-cell-derived iPSCs more readily generate insulin-producing cells, blood-derived lines produce more haematopoietic colonies, whereas fibroblast-derived iPSCs show poor haematopoietic output and stronger osteogenesis (Kim et al., 2010; Bar-Nur et al., 2011). Reprogramming also introduces reproducible aberrant methylation that persist at many loci and can impair developmental potential (Lister et al., 2011; Panopoulos et al., 2017; Ruiz et al., 2012). Large-cohort studies show that inter-individual genetic background is the dominant axis of variance: lines cluster by donor for gene expression and DNA methylation, and donor effects outweigh cell-of-origin memory in predicting differentiation propensity (Burrows et al., 2016; Kilpinen et al., 2017; Kyttala et al., 2016; Carcamo-Orive et al., 2017). However, indefinite culture of iPSCs from a single donor line should avoid this and indeed, has more potential than adult stem cell sources, although the mutational burden and within-donor variations remain. Whether–and to what extent–these donor and clone-level differences are proportionately reflected in iPSC-EV cargo and potency remains unclear; rigorous head-to-head, multi-donor EV comparisons using harmonised isolation, dose metrics and functional readouts remain scarce.

Among the few available, Turner et al. profiled EVs from human iPSC-derived cardiomyocytes generated from six donors (three with left-ventricular hypertrophy (LVH), three with normal left-ventricular mass). The miRNA composition differed by group and these differences translated into donor-specific endothelial phenotypes (tube formation, migration and proliferation) (Turner et al., 2021). However, because this design contrasts a group with pathology to those without and each arm included only n = 3 lines, it offers limited resolution on within-healthy donor variability, underscoring the need for larger multi-donor studies. By contrast, cell-state effects on EVs are better documented. For example, during iPSC-to-cardiomyocyte differentiation, EV small-RNA profiles shift in a stage-resolved manner, and EVs from the pluripotent state show stronger pro-angiogenic and pro-proliferative activity *in vitro* than EVs from committed progenitors or cardiomyocytes (Louro et al., 2022).

These donor and cell state variations remain a challenge for manufacturing. Progress in reprogramming fidelity, lineage specification and compliant EV bioprocessing - coupled with head-to-head, multi-donor studies that standardize EV characterization, dosing metrics and shared efficacy readouts - should reduce variability and support scalable, renewable iPSC-EV therapeutics (Song et al., 2024).

### 2.3 Embryonic stem cells (ESCs)

Embryonic stem cells (ESCs) represent a developmentally unrestricted source of pluripotent cells, derived directly from the inner cell mass of the preimplantation blastocyst from donated human embryos (Evans and Kaufman, 1981). Unlike iPSCs, they are not influenced by donor-cell epigenetic memory and retain broad differentiation potential (Edwards et al., 2024). They can be propagated in an undifferentiated state for extended periods under defined conditions, although culture adaptation can arise over time. Most notably, recurrent gain of chromosome 20q11.21, which increases BCL2L1/BCL-XL dosage, confers an apoptosis-resistant growth advantage, permitting variant subclones to overtake cultures (Avery et al., 2013). These lines have been linked to malignancy, although whether this transfers to EV bioactivity has not been explored (Avery et al., 2013). Routine genomic quality control, including targeted screening for 20q11.21, is therefore recommended (Avery et al., 2013; Xu et al., 2005; Rao and Zandstra, 2005; Schatten et al., 2005).

Although systematic, multi-line profiling is still limited, available datasets indicate a recurring small-RNA signal in ESC-derived EVs: across human pluripotent sources (including hESCs), EVs are enriched for the miR-302/367, miR-371/373 and miR-17-92 families linked to pluripotency, reprogramming and early developmental pathways (Kaur et al., 2018; Bi et al., 2022). Functionally, ESC EVs have been demonstrated to maintain the pluripotent state; classic work showed ESC-EVs transfer mRNAs and proteins that reprogram hematopoietic progenitors - an early demonstration of horizontal information transfer (Ratajczak et al., 2006). More recently, EV-bound fibronectin has been shown to engage integrins to activate FAK signaling in ESCs, preserving stemness gene expression and chimera-forming capacity even under differentiating conditions (Hur et al., 2021).

Evidence also indicates therapeutic promise in preclinical models. In a murine myocardial infarction model, ESC–derived exosomes were injected intramyocardially immediately after infarction and improved left-ventricular function at 4 weeks, with higher ejection fraction and fractional shortening than controls (Khan et al., 2015). Histology corroborated these functional gains with a smaller infarct, greater capillary density, and fewer apoptotic nuclei. The number of c-kit–positive cardiac progenitor cells (CPCs) also rose by approximately 2.5-fold, consistent with enhanced survival and proliferation of endogenous progenitors. Mechanistic probing implicated miR-294, which promoted S-phase entry in CPCs *in vitro*, although *in vivo* necessity and dose–response relationships remain undefined (Khan et al., 2015). It should be noted that many foundational studies, including this one, normalized vesicle input by bulk protein rather than by particle number (for example, nanoparticles-per-milliliter measured by nanoparticle tracking analysis) (Lai et al., 2010; Khan et al., 2015). Protein-based dosing can vary with co-isolated protein contaminants and with shifts in the protein-to-particle ratio across isolation methods, complicating potency assignment and cross-study comparison. Future studies should report particle counts alongside protein and use particle-normalized (or EV-marker-normalized) dosing with transparent characterization, so that effective dose, mechanism, and reproducibility can be evaluated rigorously (Welsh et al., 2024).

Owing to their unrestricted developmental potential and stable proliferative capacity, ESCs arguably provide the most biologically robust source of regenerative EVs. Yet clinical use is constrained by ethics and law. In the UK, for instance, research on human embryos is permitted only under an HFEA research license and only up to 14 days after fertilization; embryos are typically surplus IVF embryos donated with consent, and research embryos may not be transferred (Human Fertilisation and Embryology Authority, 2025; Human Fertilisation and Embryology Act, 1990). In the USA, hESC research is lawful, but federal funds cannot be used to derive new lines under the Dickey–Wicker Amendment, so NIH-funded work is limited to approved lines that meet consent and provenance requirements (NIH, 2021; Dickey–Wicker Amendment, 1996). In Germany, domestic derivation of hESCs is prohibited; research is restricted to imported lines under strict conditions (EuroStemCell, 2017a). In Italy, research on human embryos is prohibited under Law 40/2004, except for procedures that are strictly intended to protect the health or development of the embryo itself (EuroStemCell, 2017b). These frameworks limit not only cell-based products but also the sourcing, banking and clinical development of ESC-EVs (Lo and Parham, 2009).

### 2.4 Hematopoietic stem cells (HSCs)

Compared with MSCs and iPSCs, hematopoietic stem cells are more lineage restricted, with differentiation largely confined to blood lineages (Osawa et al., 1996; Notta et al., 2016). They are classically defined as CD34 positive, CD38 negative, lineage negative cells with long term repopulating capacity *in vivo*, a functional criterion that keeps them distinct from the broader pool of early hematopoietic progenitors. Early claims of broad plasticity in non-hematopoietic tissues were re-evaluated by genetic fate-mapping, which showed that apparent contributions in liver, brain and heart mostly reflected cell fusion rather than true transdifferentiation, indicating any such plasticity is rare and context dependent (Balsam et al., 2004; Alvarez-Dolado et al., 2003; Wagers et al., 2002).

Within the EV literature, proangiogenic activity of HSC-derived EVs is the most referenced (Ratajczak et al., 2013; Sahoo et al., 2011). Cord blood, although containing a lower proportion of HSCs than bone marrow aspirates, provides a notably ‘youthful’ HSC and progenitor pool, which is one reason it has become an attractive starting material for EV production. Using umbilical cord blood (UCB)–derived CD133 enriched early progenitors, microvesicle-enriched fractions were shown to chemoattract human umbilical-vein endothelial cells (HUVECs) and to increase their tube formation in multiple *in vitro* assays, as well as to harbor angiogenic-related mRNAs, although the qPCR was targeted and “enrichment” was defined relative to bulk UCB mononuclear cells, not to CD133^-^ fractions or endothelial comparators, which limits interpretability. *In vivo*, they report greater hemoglobin content in subcutaneous Matrigel plugs in SCID mice after CD133^+^ MVs (versus empty plugs and platelet MVs), but provide only small-n summary values from two experiments (n = 3/group) without images or statistical testing; accordingly, the evidence for pro-angiogenic activity *in vivo* is suggestive rather than compelling (Ratajczak et al., 2013). More convincingly, exosomes purified from mobilized human CD34-positive cells (40–90 nm; CD63/TSG101-positive) recapitulated the parent cells’ paracrine effects, enhancing endothelial viability, proliferation and tube formation *in vitro*. *In vivo*, they promoted neovascularization: subcutaneous Matrigel plugs containing exosomes and corneal implants with exosome pellets showed greater vessel length than PBS or exosomes from CD34-depleted mononuclear cells (Sahoo et al., 2011). Extending this to ischemia, intramuscular delivery of human CD34-cell exosomes into the ischemic hindlimb improved perfusion and capillary density, and knockdown of miR-126 - an endothelial-enriched microRNA that augments VEGF signaling - in donor cells abrogated these benefits, establishing a cargo–function link (Mathiyalagan et al., 2017; Fish et al., 2008).

Of note, most EV studies analyze vesicles from bulk CD34^+^ or CD133^+^ cord-blood fractions, which are heterogeneous and include committed progenitors. By contrast, long-term HSC activity is enriched within Lin^−^CD34^+^CD38^−^CD90^+^CD45RA^−^ (and further within CD49f^+^), indicating that bulk CD34^+^/CD133^+^ populations only partially overlap the true LT-HSC pool. Consequently, findings attributed to ‘HSC-EVs’ from bulk CD34^+^/CD133^+^ sources are best interpreted as originating from mixed HSPCs rather than purified LT-HSCs (Majeti et al., 2007; Notta et al., 2011).

Clinical translation of HSC-EVs is constrained by scale, because authentic human long-term HSCs expand poorly *ex vivo;* even small-molecule approaches (such as the aryl-hydrocarbon–receptor antagonist SR1) only partially relieve this bottleneck (Boitano et al., 2010). To address supply constraints, continuous leukemic progenitor cell lines can provide scalable yields of EVs. However, EVs from the human acute myeloid leukemia (AML) line, KG-1a, carry E-selectin ligands, bind E-selectin *in vitro*, and accumulate in spleen and spine *in vivo* in an E-selectin-dependent manner, indicating disease-imprinted cargo and endothelial targeting. Moreover, although both KG-1a and healthy CD34^+^ HSPC exosomes share adhesion/migration programs, the AML exosome datasets additionally map to tumor-relevant signaling - so they are unlikely to be useful surrogates for clinical HSC-EVs (Isaioglou et al., 2023). Comparative, multi-donor studies are needed to quantify leukemia-versus-normal differences and to link specific cargo to function (Isaioglou et al., 2023). Recent biomimetic “microniche” systems have expanded functional subsets of human HSCs, suggesting primary sources may become more practical as manufacturing improves (Li et al., 2023a); however, unlike pluripotent platforms with renewable starting material, HSC-EV applications are likely to remain more niche until potency and scalability are proven. Similar scarcity of rigorous multi-donor and batch-to-batch studies on purified HSC/HSPC-EVs - using harmonized isolation and particle-normalized dosing - limit clinical translation, but unique to the HSC-EV is the restricted differentiation and therefore, more narrow clinical application.

### 2.5 Trophoblast stem cells (TSCs)

Trophoblast stem cells (TSCs) represent a comparatively underexplored source of EVs with distinct immunological and regenerative profiles (Okae et al., 2018). Unlike ESCs, which are pluripotent derivatives of the inner cell mass, trophoblast stem cells arise from the trophectoderm and are restricted to generating the fetal component of the placenta and are integral to mediating maternal–fetal immune tolerance (Okae et al., 2018). Human TSC lines can be established from blastocysts or first-trimester cytotrophoblasts (placental villi) and maintained long-term (∼85-150 population doublings), enabling scalable production compared with adult stem-cell sources (Okae et al., 2018). Notably, the same has not been achieved from term placentas (Okae et al., 2018). Early, first trimester, human TSCs have also been isolated from chorionic villi of ectopic pregnancies - clinically discarded pre-placental tissue obtained with consent - offering an early, ethically accessible source (Jau-Nan Lee and Lee, 2018).

A distinctive property of trophoblasts is their innate immune modulation at the maternal–fetal interface. Once uteroplacental circulation is established and maternal blood bathes the chorionic villi, maternal leukocytes directly encounter semi-allogeneic trophoblasts, necessitating local mechanisms that avert rejection (PrabhuDas et al., 2015). Extravillous trophoblast (EVT) constitutively expresses HLA-G, a non-classical MHC class I molecule that promotes maternal–fetal tolerance by limiting activation of decidual natural killer (dNK) cells at the implantation site (Tilburgs et al., 2015). Interestingly, EVTs can transfer HLA-G to dNK cells via contact-dependent uptake, dampening dNK cytotoxicity; conversely, inflammatory cytokines induce HLA-G degradation within dNK cells, restoring cytotoxic function - an “HLA-G cycle” that toggles between tolerance and antiviral defense (Tilburgs et al., 2015). Complementing this, placental explants release exosomes bearing NKG2D ligands that downregulate NKG2D receptor expression on NK, CD8^+^ and γδ T cells, reducing their cytotoxicity *in vitro* and providing direct evidence that placental EVs can suppress maternal effector responses (Hedlund et al., 2009).

Much of this immune modulation has been shown to be mediated in a paracrine manner through the release of EVs (Stenqvist et al., 2013; Delorme-Axford et al., 2013; Bai et al., 2022). Primary human trophoblast (PHT) exosomes are enriched for C19MC microRNAs, with small-RNA profiling showing that these miRNAs predominate in the vesicle cargo and mirror cellular expression (Donker et al., 2012). Functionally, trophoblast conditioned medium and purified PHT exosomes delivered C19MC miRNAs to non-trophoblast recipient cells and lowered infection rates and viral replication across vesicular stomatitis virus, coxsackievirus B, vaccinia, HSV-1, HCV and CMV; blocking autophagy with 3-methyladenine or genetic disruption reduced these effects (Delorme-Axford et al., 2013). Such anti-viral properties of PHT exosomes, and indeed of C19MC miRNAs independently, have been replicated across multiple studies (Ouyang et al., 2016; Bayer et al., 2018). However, these findings are confined to *in vitro* assays, and protective effects *in vivo* are yet to be demonstrated.

Beyond antiviral and immune-modulatory effects, a recent study has now provided direct evidence that human trophoblast stem cell secretome and EVs from ectopic pregnancies can attenuate senescence-associated phenotypes *in vitro* (Abdelmohsen et al., 2026). Using irradiated or etoposide-treated WI-38 fibroblasts, Abdelmohsen et al. showed that hTSC-conditioned medium, and particularly purified hTSC-EVs, reduced the expression and secretion of canonical SASP factors including CXCL1, IL8 and GDF15, while also lowering DNA-damage signaling and NF-kB activation. Proteomic analysis indicated enrichment of extracellular-matrix, adhesion and tissue-remodeling proteins in the hTSC secretome and EV fraction, consistent with a broader reparative program. Importantly, this study used human trophoblast stem cells rather than an immortalized EVT-like surrogate, indicating senomorphic potential. Nevertheless, this evidence also remains confined to *in vitro* fibroblast models, and warrants investigations into *in vivo* efficacy (Abdelmohsen et al., 2026).

Other preclinical data on TSC-EVs remain sparse, though hTSCs themselves have been shown to closely resemble primary trophoblasts at the transcriptome and methylome level, supporting their relevance as a model (Okae et al., 2018). Functionally, one study reported that EVs isolated from “TSC” cultures enhanced MSC proliferation, reduced senescence, and promoted osteogenic differentiation *in vitro* (Go et al., 2023). The authors also profiled EV cargo at both the miRNA and mRNA levels, adding mechanistic plausibility to these effects. These effects were then recapitulated *in vivo*, as MSCs preconditioned with these EVs achieved greater wound closure in a murine full-thickness skin-defect model and enhanced bone regeneration in a rat calvarial-defect model compared with MSCs alone and MSCs pretreated with MSC-EVs (Go et al., 2023). While certainly interesting, these results should be considered with two following cautions: first, as discussed earlier, donor non-matching introduces substantial variability, making it difficult to apportion effects to EV biology versus inter-donor differences; second - the “TSC” producer in that study was the immortalized EVT-like line HTR-8/SVneo, not *bona fide* hTSCs, so the findings reflect immortalized EVT biology rather than stem-cell–state EV function (Go et al., 2023). In separate experiments, trophoblast-derived EVs and conditioned medium increased human dermal fibroblast proliferation and migration in a dose- and time-dependent manner, with associated upregulation of extracellular-matrix genes and downregulation of NF-κB signaling (Go et al., 2021). Similarly, in lipopolysaccharide-injured human middle-ear epithelial cells, trophoblast conditioned medium and EVs improved cell viability and suppressed inflammatory gene expression via COMMD1-linked modulation of NF-κB (Lee et al., 2023a). To our knowledge, the same effects have not been investigated *in vivo*. While TSCs offer clear practical advantages for EV manufacture - renewable lines established under defined conditions and ethically sourced tissues compared with embryonic stem cells - the therapeutic evidence base is still very early. Rigorous comparative studies with other stem-cell EV sources, using harmonized isolation methods, particle-normalized dosing, and shared functional assays, will be essential to establish potency, scalability, and clinical value (Go et al., 2023).

### 2.6 Comparative studies

There are surprisingly few direct head-to-head comparisons of EV sources. Two studies that compare iPSC-EVs with MSC-EVs suggest an efficacy edge for iPSC-EVs in epithelial and cartilage repair. In a rat corneal epithelial defect model, topical iPSC-EVs and MSC-EVs (both isolated by ultracentrifugation) accelerated re-epithelialization versus vehicle, with iPSC-EVs producing significantly faster closure *in vivo* and stronger pro-proliferative, pro-migratory, and anti-apoptotic effects on human corneal epithelial cells *in vitro* under identical dosing conditions (Wang et al., 2020b). In collagenase-induced murine osteoarthritis, intra-articular iPSC-MSC–EVs (iMSC-EVs) and synovial-MSC EVs both improved joint structure and chondrocyte behavior; however, iMSC-EVs OARSI histological scores were significantly lower than synovial-MSC-EV-treated mice, and they elicited greater chondrocyte migration and proliferation *in vitro* (Zhu et al., 2017).

A broader, multi-arm cardiac study compared small EVs prepared under a common workflow from human ESCs, ESC-derived cardiac progenitors and cardiomyocytes, primary BM-MSCs, hTERT-immortalized MSCs, and ventricular cardiac fibroblasts. ESC-EVs consistently ranked best *in vitro* for pro-angiogenic and anti-fibrotic activity and, in a mouse myocardial ischemia–reperfusion model, yielded the most favorable *in vivo* outcome (less adverse remodeling with increased angiogenesis and reduced fibrosis) under matched dosing. Omics profiling showed that ESC-EV cargo clustered more closely with that of ESC-derived cardiac progenitors than with MSC-EVs, but the study did not ascribe the functional advantage to a single molecule or pathway (Gonzalez-King et al., 2024). Complementing this, a cargo-only comparison of exosomes from hESCs, hiPSCs and umbilical-cord MSCs (ultracentrifugation +0.22-μm filtration) reported pathway-level differences in protein/miRNA signatures suggestive of stronger “developmental/metabolic” programs in pluripotent-cell exosomes and more immune-modulatory signatures in UC-MSC exosomes; however, there was no functional validation, so these inferences should be treated as hypothesis-generating rather than conclusive (Bi et al., 2022).

Critically, all these comparisons drew on different donors for the cell sources being contrasted. As noted earlier, donor origin is a major driver of EV compositional variability; it could plausibly explain, for example, why ESC-derived cardiomyocyte EVs clustered more closely with other ESC-lineage EVs than with MSC-EVs in the cardiac study (Gonzalez-King et al., 2024). Because donor effects were not controlled, we cannot exclude the possibility that younger/healthier MSC donors, or matched-donor designs, might have shifted outcomes. The most rigorous approach would compare iPSCs and their isogenic iMSCs derived from the same donor; notably, this was not done in the studies above. More generally, larger designs with multiple donors per group, harmonized isolation, particle-normalized dosing and shared readouts are required, especially given the small n typical of the animal experiments cited. Until such data accrue, claims of source “superiority” should remain indication-specific and model-bounded rather than general. Finally, no published head-to-head studies have yet included trophoblast-stem-cell EVs under harmonized conditions - an obvious gap for the field.

## 3 Mechanistic actions of EVs in aging and regeneration

Stem cell-derived EVs exert their effects by transferring protected and biologically active cargo, including miRNAs, mRNAs, proteins, lipids, and metabolites, that can reprogram intracellular signaling and reshape the extracellular environment (Lapasset et al., 2011). Their actions have been shown to converge on several recognized hallmarks of aging, notably chronic inflammation, cellular senescence, impaired intercellular communication, and loss of regenerative capacity. However, the mechanisms by which they do this are poorly understood. In particular, many distribution studies show an immediate accumulation of EVs at the site of the liver, notably a clearing organ. Nonetheless, many preclinical models have demonstrated efficacy in models of inflammatory disease, tissue repair and angiogenesis, cellular senescence and neurodegeneration (Khan et al., 2015; Losurdo et al., 2020; Willis et al., 2018; Mathiyalagan et al., 2017; Zhou et al., 2018; Dorronsoro et al., 2021; Fang et al., 2023). Evidence for this will be discussed in turn, below.

### 3.1 Immunomodulation and inflammatory resolution

Chronic, low-grade inflammation (“inflammaging”) is a hallmark of aging and age-related diseases (Lopez-Otin et al., 2023). EVs derived from mesenchymal stem cells have been shown to promote anti-inflammatory signaling across many models of inflammatory disease, including arthritis, sepsis, cardiovascular disease, and neurological conditions (Lai et al., 2010; Lee et al., 2023a; Li et al., 2025; Cosenza et al., 2018; Song et al., 2017; Chen et al., 2020), For instance, an extensive study by Phinney et al. (2015) demonstrated that MSCs respond to oxidative stress by exporting depolarized mitochondria in microvesicles and, in parallel, releasing small EVs enriched in immunomodulatory microRNAs, notably miR-451 (Phinney et al., 2015). This was evidenced by electron and live-cell confocal imaging showing mitochondria packaged into LC3/ARRDC1-positive membrane blebs that bud as microvesicles; mouse macrophages phagocytosed these vesicles (uptake blockable by dextran sulfate) and retained donor mitochondrial fluorescence and human mitochondrial DNA, with dual-color labelling demonstrating fusion of transferred mitochondria with the host network. Functionally, macrophages exposed to MSCs or their exosomes showed improved bioenergetics on Seahorse analysis (higher oxygen-consumption rates with reduced proton leak) and were protected against silica-induced mitochondrial ROS and respiratory collapse. In parallel, small EVs carried a distinct miRNA cargo enriched for miR-451, and exosome uptake reprogrammed TLR/NF-κB signaling with reduced pro-inflammatory cytokine output. Consistent with these mechanisms, a single intravenous dose of MSCs or their EVs mitigated silica-induced lung inflammation and fibrosis in mice, lowering inflammatory cell influx, cytokines and collagen deposition (Phinney et al., 2015). Extending these *in vivo* results, several studies demonstrate therapeutic efficacy of MSC-EVs in various models of Acute Lung Injury (ALI), including intratracheal silica and LPS challenges (Hou et al., 2023; Zhao et al., 2022; Bandeira et al., 2018). For instance, in an LPS-induced murine ALI model, a single inhaled dose of MSC-EVs (50 μg in 50 μL, delivered 3 h after LPS) reduced circulating IL-1β, IL-1α, MCP-1, TNF-α and IL-12 while increasing IL-10, with concordant improvements in lung histopathology versus vehicle (Zhao et al., 2022).

Extending these findings, several murine cecal ligation and puncture (CLP) studies show that systemically administered stem-cell-derived EVs improve survival, reduce circulating inflammatory cytokines, and preserve organ function (Zhou et al., 2018; Song et al., 2017; Wang et al., 2015a; Wang et al., 2022a; Yang et al., 2023). For example, in CLP, a single intravenous dose of endothelial progenitor cell (EPC) exosomes - 2 mg protein/kg given 4 h post-CLP - lowered plasma interleukin-6, interferon-γ, tumor necrosis factor-α and monocyte chemoattractant protein-1, increased interleukin-10, limited lung and renal vascular leak, and improved 7-day survival; knockdown of exosomal miR-126-3p/-5p abrogated these effects (Zhou et al., 2018). Notably, across CLP studies the proposed mediators differ - benefits have been variously attributed to exosomal miR-146a, miR-223, or miR-126 - so a single unifying mechanism has yet to emerge (Zhou et al., 2018; Song et al., 2017; Wang et al., 2015a; Wang et al., 2022a; Yang et al., 2023).

### 3.2 Tissue repair

Moving from inflammaging to regeneration, preclinical studies show that stem cell–derived EVs can actively drive tissue repair in animal models and *in vitro* (Abolgheit et al., 2021; Shi et al., 2017; Ma et al., 2021; Liu et al., 2022; Zhang et al., 2015a). For example, in a rat full-thickness cutaneous wound model (three 18-mm dorsal excisions), human iPSC-MSC exosomes were delivered peri-wound by subcutaneous injection (dosed by protein mass; 160 µg in 160 µL across four sites) with an additional 40 µg applied to the wound bed; this regimen produced significantly greater wound closure at days 4, 7 and 14, increased percentage re-epithelialization and narrower scar width at day 14, and more mature collagen by Masson’s trichrome compared with vehicle controls (Zhang et al., 2015b). Neovascularization was also enhanced, quantified by higher densities of CD31-positive vessels and increased numbers of CD31/α-SMA double-positive mature vessels at days 7 and 14. Complementary *in-vitro* assays showed dose-dependent pro-repair activity: human dermal fibroblasts exhibited increased proliferation, faster scratch-wound migration (approximately 3-fold at 12 h and 2-fold at 24 h with 50–100 μg/mL), elevated fibronectin, and higher secretion and mRNA expression of type I/III collagen and elastin; human umbilical vein endothelial cells displayed increased proliferation, migration and tube formation (greater total tube length and branch points) (Zhang et al., 2015b). Together, these findings provide concrete evidence - across *in vivo* rat skin repair and aligned *in-vitro* human cell readouts - that iPSC-MSC exosomes accelerate re-epithelialization, angiogenesis and matrix maturation in cutaneous wound healing.

Similar findings have been demonstrated with MSC-EVs from umbilical cord and adipose tissue. In a rat deep second-degree burn model, exosomes from human umbilical-cord MSCs enhanced endothelial proliferation, migration and tube formation *in vitro* and improved wound healing *in vivo* via a Wnt4→β-catenin program; blocking β-catenin signaling or knocking down Wnt4 abolished both the pro-angiogenic and reparative effects (Zhang et al., 2015c). Complementing this, adipose-derived MSC-EVs significantly accelerated closure of murine dorsal excisional wounds after either local or intravenous administration, with *in vivo* tracking showing EV accumulation at the wound and fibroblast assays demonstrating increased proliferation, migration and early collagen I/III production consistent with expedited repair (Hu et al., 2020). Beyond speed, adipose-exosome treatment also improved the quality of healing in mouse incisional wounds, reducing scar size and shifting matrix remodeling toward a low-scarring phenotype - higher collagen III:I and TGF-β3:TGF-β1 ratios, higher MMP3:TIMP1, and restraint of myofibroblast (α-SMA) differentiation (Wang et al., 2021).

### 3.3 Modulation of cellular senescence and the SASP

Cellular senescence is a hallmark of aging characterized by a stable cell cycle arrest accompanied by the acquisition of a pro-inflammatory, tissue-remodeling secretome termed the senescence-associated secretory phenotype (SASP) (Coppe et al., 2008). Senescent cells accumulate in aging tissues and contribute to local and systemic dysfunction by impairing regeneration, promoting chronic inflammation, and degrading extracellular matrix components (Lopez-Otin et al., 2013). Emerging evidence suggests that stem cell-derived EVs, both embryonic and adult, can modulate senescence both by acting directly on senescent cells and by altering the surrounding tissue microenvironment (Zhang et al., 2025; Boulestreau et al., 2020). For instance, treatment of late-passage mouse embryonic fibroblasts with ESC–derived EVs (100 μg/mL EVs for 96 h) reduced characteristic senescence markers, including SA-β-gal, p21, p53, and reactive oxygen species (Yu et al., 2023). These findings were extended *in vivo*; aged C57BL/6 mice (14 months) receiving intraperitoneal injections of 100 μg ESC-EVs every 2 days for 8 weeks had reduced SA-β-gal positivity in liver, kidney and spleen, as well as decreased kidney and liver p16, p21, p53, IL1a and IL11 and corresponding improved hepatic and renal histology (Yu et al., 2023). miRNA sequencing of the ESC-EVs was performed to probe mechanistic pathways and compared to that of Mouse Embryonic Fibroblast (MEF)-EVs as controls. Of the 238 differentially expressed miRNAs, the authors identified those most related to senescence pathways. While this reflects a targeted hypothesis on senescence, given the authors performed an untargeted miRNA-seq, it would have been interesting to additionally evaluate the most enriched miRNAs. Nevertheless, overexpression of a selected group of senescence-related miRNAs in P7 MEFs (passage 7) significantly decreased the aforementioned senescence markers, and inhibition (specifically of miR-15b-5p and miR-290a-5p) reversed this, suggesting these miRNAs are sufficient to recapitulate the ESC-EV anti-senescence activity (Yu et al., 2023). Complementing this, purified iPSC-derived EVs applied to senescent MSCs in culture decreased ROS, SA-β-gal and p21/p53, as well as SASP cytokines, providing further *in vitro* evidence but with human cell lines (Liu et al., 2019). Concordant vascular evidence comes from MSC-EVs that reduced senescence biomarkers in senescent HUVECs and improved wound healing in aged and diabetic mice, as demonstrated by smaller wound areas and greater re-epithelialization at 12 days post-surgery in mice that received subcutaneous injection of MSC-EVs around the wound at the time of surgery (Xiao et al., 2021). Whole miRNAome analysis of both the MSC-EVs and the recipient HUVECs identified only 4 overlapping miRNAs and inhibition of one of these, miR-146a (out of two tested) abolished these *in vitro* MSC-EV effects, leading the authors to attribute the effects to this particular miRNA. Notably, the same investigations were not performed *in vivo* (Xiao et al., 2021).

### 3.4 Central nervous system disorders

Among CNS disorders, the therapeutic efficacy of EVs in stroke has been widely modelled across research groups (Xin et al., 2013; Doeppner et al., 2015; Zhang et al., 2021). In a comparative study, EVs derived from bone marrow mesenchymal stem cells (BMSC-EVs) and brain endothelial cells were administered following permanent middle cerebral artery occlusion (pMCAO) in rats. Both EV types attenuated BBB breakdown by downregulating Caveolin-1 (Cav-1) – a key mediator of tight junction protein endocytosis and degradation–and concurrently upregulated ZO-1 and Claudin-5 expression within 24 h of stroke onset. Notably, BMSC-EVs were more effective than brain endothelial EVs in suppressing Cav-1 expression and improving neurological outcomes (Li et al., 2023b).

In addition to direct effects on endothelial cells, EVs have been shown to exert neuroprotective actions by modulating glial cell activity. For example, immunohistological techniques have been used to demonstrate MSC-EV-mediated reductions in gliosis following LPS-induced brain injury in rats (Drommelschmidt et al., 2017). Numerous reports have also highlighted their therapeutic potential in rodent models of spinal cord injury (SCI) (Rong et al., 2019; Romanelli et al., 2019; Ruppert et al., 2018). For example, in a severe contusive rat SCI model, a subdural porous microneedle patch carrying a GelMA hydrogel seeded with mesenchymal stem cells was placed over the lesion, so that the embedded cells continuously secreted extracellular vesicles that diffused through the microneedles for at least 7 days. This sustained, local EV delivery reduced cystic cavitation and glial scar, increased vascular density, preserved axons, and improved locomotor function, outperforming patches loaded with EVs alone or systemic or local MSC delivery without the patch, which underscores how dosing kinetics and precise localization govern efficacy (Fang et al., 2023). Clearly, the clinical translation of this device is complicated compared to an acellular product.

Other delivery strategies are also being explored. For example, in a murine model of controlled cortical impact (CCI)-induced TBI, intranasal administration of human MSC-derived EVs 90 min post-injury was shown to reduce the acute neuroinflammatory response, as evidenced by reduced expression of NLRP3 inflammasome components (NLRP3, IL-1β, and IL-18), and thereby prevent chronic activation of the NLRP3–p38/MAPK axis. Importantly, animals treated with MSC-EVs demonstrated improved cognitive and affective behaviors compared to vehicle-treated controls, indicating long-term neuroprotective benefits (Kodali et al., 2023). Together, these findings suggest that stem cell-derived EVs may preserve BBB integrity and neural architecture by suppressing glial activation, reducing neuroinflammatory signaling, and sustaining synaptic function. These mechanisms are of particular interest in the context of aging, in which chronic neuroinflammation and barrier breakdown are implicated in the pathogenesis of cognitive decline and cerebrovascular dysfunction (Zhang et al., 2021; Apodaca et al., 2021). Clinical translation, however, is far from straightforward. In stroke and TBI or SCI, heterogeneity in lesion type, timing, and standard-of-care interventions complicates trial design, blunts effect sizes, and forces narrow enrolment windows that are hard to meet (Furlan, 2012; Ma et al., 2022; Krishna et al., 2014). Of course, targeting aging presents yet more hurdles. It is not classified as a disease, so there is no single diagnostic entry point, no agreed primary endpoint, and no regulatory framework tailored to preventing or slowing physiological decline (Justice et al., 2016). The next section examines the specific hurdles for EVs in this landscape, focusing on sources of variability in EV composition and activity that must be overcome for clinical utility.

## 4 Factors influencing the quality and function of stem cell-derived EVs

The therapeutic efficacy of EVs is strongly influenced by the biological characteristics of the parent cells. Donor age, passage number, environmental stressors, and epigenetic memory can all affect the yield, composition, and bioactivity of EVs. Understanding and controlling these variables is critical for ensuring consistency, potency, and safety in clinical applications (Taylor and Shah, 2015; Mushahary et al., 2018; Alibhai et al., 2020; Abbasi Sourki et al., 2023; Nguyen et al., 2020). In practice, such lot to lot and donor to donor variability means that ostensibly the same therapy may deliver compositionally different vesicles to different patients, which undermines regulatory equivalence, muddies dose response and safety attribution, and risks inconsistent efficacy or even harm in susceptible contexts. EVs ultimately mirror the physiological state of the parent cells: senescence and stress remodel EV quantity and cargo, including miRNAs and proteins, with functional consequences for regenerative potency (Palama et al., 2025; Terlecki-Zaniewicz et al., 2018). Herein, we discuss the relevant factors influencing the quality and function of EVs.

### 4.1 Replicative senescence and In vitro aging of donor cells

The *in vitro* aging of stem cells, which is commonly referred to as replicative senescence, has a significant impact on the quality, cargo composition, and therapeutic efficacy of derived EVs (Fafian-Labo et al., 2021; Fafian-Labora et al., 2020). Replicative senescence, a phenomenon driven by telomere shortening and cumulative cellular stress during serial passaging (the Hayflick limit), leads to irreversible growth arrest and functional decline (Hayflick and Moorhead, 1961). Because adult MSCs begin with shorter telomeres and lower telomerase activity, they reach senescence after fewer doublings *in vitro*, making them particularly susceptible to early exhaustion (Lupatov and Yarygin, 2022).

Late-passage MSCs undergo asymmetric division, with one daughter cell entering a senescent state characterized by G1 cell cycle arrest, flattened morphology, and increased activity of senescence-associated β-galactosidase (SA-β-gal) and lysosomal α-L-fucosidase (SA-α-Fuc) (Campisi and d'Adda di Fagagna, 2007). These cells also exhibit increased cytoskeletal tension, reduced adherence, and cytoplasmic granularity, along with heterogeneous surface marker expression, differentiation potential, and immunophenotype (Schellenberg et al., 2012). As a result, the EVs produced from senescent MSCs are more heterogeneous and less predictable in function, undermining consistency across therapeutic applications. This leads to inconsistent results with therapeutic interventions using MSC-derived exosomes, especially for slowing or reversing age-related degenerative changes or enhancing the tissue repair process (Schellenberg et al., 2012).

Senescence alters EV output both quantitatively and qualitatively (Alibhai et al., 2020). Senescent MSCs tend to secrete greater numbers of EVs, but these are typically smaller in size and altered in content–especially microRNAs and other regulatory RNAs, as shown by transcriptomic profiling (Lei et al., 2017). The cargo of these EVs reflects the senescent state of the parent cells, often resulting in diminished regenerative capacity. For example, microvesicles derived from senescent cells have shown reduced efficacy in promoting tissue repair (Liu et al., 2020b). Consequently, effective clinical and cosmetic use of stem cell-derived EVs requires careful consideration of donor cell passage number, senescence status, and the broader impacts of *in vitro* culture conditions on EV quality and potency.

One possible way to overcome such a limitation is via the use of iMSCs, which are reprogrammed to a youthful telomere and epigenetic state, can be clonally banked, and expanded at scale under defined conditions, thereby reducing donor-related drift and early-passage senescence (Lee et al., 2023b). However, even when derived from a single iPSC line using comparable differentiation and culture conditions, independent production runs still diverge in EV cargo and functional potency. In GMP-style iPSC-MSC workflows, this variability arises primarily from passage-dependent changes in cell quality, subtle differences in differentiation efficiency and metabolic state between batches, and small process-related fluctuations in culture microenvironment or EV isolation purity (Tertel et al., 2023). These factors manifest as batch-to-batch differences in protein profiles and immunosuppressive efficacy in mdMLR assays, while parallel studies report variable anti-inflammatory activity across separate iMSC differentiation batches in osteoarthritis co-culture models (Palama et al., 2025). Such findings indicate that iMSC sourcing attenuates replicative aging but does not eliminate variability, so feeder-free, chemically defined production with batch-level potency assays, quantitative dose normalization, and mechanism-linked release criteria remains essential (Palama et al., 2025; Tertel et al., 2023).

### 4.2 Donor age and telomere length

Donor age is a major determinant of stem-cell function and EV quality (Fichtel et al., 2022; Boulestreau et al., 2020). As organisms age, stem cells exhibit cumulative metabolic stress, oxidative damage, and epigenetic remodeling that together erode their regenerative potential (Liu et al., 2020b; Beerman and Rossi, 2014). In both rodents and humans, EVs derived from aged donors display altered composition and diminished biological efficacy (Wang et al., 2015b; Huang et al., 2019). For example, intravenous administration of serum-derived EVs from young mice reduces systemic “inflammaging”, lowering IL-6, IL-1β, and splenic TNF-α levels (Wang et al., 2018), whereas EVs from peripheral blood of aged mice (24 months) induce glial activation and upregulate brain GFAP and CD68 in young recipients (Morales-Prieto et al., 2022). Similarly, bone-marrow EVs from aged mice (24–28 months) impair proliferation and osteogenic differentiation of young BMSCs *in vitro* (Davis et al., 2017), while EVs from young or neonatal sources improve exercise capacity, organ function, and survival in aged rats (Grigorian et al., 2023). Similarly, EVs derived from young MSCs, including those generated from human ESCs, reduced markers of cellular senescence, improved health span (measured by multiple parameters including behavioural tests and circulating inflammatory mediators), and extended lifespan in murine models of natural and accelerated aging, supporting the senotherapeutic potential of youthful MSC EV cargo (Dorronsoro et al., 2021). Collectively, these findings highlight donor age as a key extrinsic variable shaping EV potency through its impact on stem-cell physiology.

Telomere attrition provides one principal molecular basis for this decline. Adult stem cells such as MSCs and HSCs possess shorter telomeres and a more limited proliferative lifespan than iPSCs, ESCs, or early TSCs, rendering them particularly susceptible to replicative senescence (Okae et al., 2018; Jau-Nan Lee and Lee, 2018; Baxter et al., 2004; Stenderup et al., 2003; Bonab et al., 2006; Agarwal et al., 2010; Allsopp et al., 2001; Amit et al., 2000). Telomere length serves as an index of cellular aging that influences stem-cell viability, differentiation potential, and the molecular composition of secreted EVs (Lei et al., 2017; Harley et al., 1990). Telomere erosion is accelerated by oxidative stress and cumulative metabolic demand (Harley et al., 1990; Richter and von Zglinicki, 2007), eliciting DNA-damage responses and transcriptional reprogramming that compromise stem-cell function and reduce the regenerative potency of their vesicles (Wagner et al., 2008; Lei et al., 2017; Estrada et al., 2013; Kim et al., 2020). iPSCs and ESCs, in contrast, maintain telomerase activity and stable telomere length, conferring a higher proliferative ceiling (Agarwal et al., 2010; Amit et al., 2000). TSCs display comparable stability: derived from first-trimester chorionic villi, they retain normal karyotype and extended telomeres through serial passages (Jau-Nan Lee and Lee, 2018). Quantitative analyses using qPCR and TRF assays confirm that telomeres are longest in first-trimester placental villi, shorten by term, yet remain longer than those in cord-blood mononuclear cells (Novakovic et al., 2016; Allsopp et al., 2007), suggesting that developmentally early stem-cell sources possess intrinsic resistance to replicative exhaustion.

Epigenetic drift operates alongside telomere shortening as a second driver of functional decline. Age-related alterations in DNA methylation and histone modifications progressively remodel chromatin, limiting transcriptional flexibility and lineage potential in stem cells (Wang et al., 2022b). In HSCs, genome-wide studies show hypermethylation of Polycomb-repressive-complex targets and redistribution of histone marks (H3K4me3, H3K27me3), correlating with myeloid bias and reduced self-renewal (Beerman and Rossi, 2014). Similarly, in human MSCs, donor-age-dependent changes in DNA methylation and hydroxymethylation occur at enhancer and developmental loci, particularly within *HOX*gene clusters, leading to diminished osteogenic and chondrogenic differentiation potential (Cakouros and Gronthos, 2020). Together, these studies demonstrate that telomere erosion and epigenetic remodeling act in concert to exhaust adult stem-cell function, progressively reducing the quality, abundance, and regenerative efficacy of their extracellular vesicles. These intrinsic, age-related processes make adult stem cells increasingly vulnerable to oxidative and environmental stressors, which further degrade stem-cell performance and the fidelity of EV production (Liu et al., 2020b).

### 4.3 Oxidative stress and environmental exposures

Building on these intrinsic aging mechanisms, oxidative stress represents a major extrinsic determinant of stem-cell function that influences EV yield, cargo composition, and therapeutic potency (Denu et al., 2016; Khanh et al., 2020; Chiaradia et al., 2021). Adult stem cells have been shown to be particularly susceptible to oxidative damage, relying on tightly balanced redox homeostasis and with a limited capacity to buffer reactive oxygen species (Orciani et al., 2010). During serial passaging, MSCs progressively accumulate oxidative stress, showing reduced superoxide dismutase (SOD) activity together with elevated levels of reactive oxygen species, nitric oxide, and oxidized or glycated proteins (Sun et al., 2024; Stolzing and Scutt, 2006; Bertolo et al., 2017). This intracellular stress not only accelerates replicative exhaustion but also perturbs EV biogenesis, as demonstrated by recent evidence that oxidative stress can activate enzymatic pathways such as β-hexosaminidase B, leading to impaired multivesicular body processing and altered exosome release and cargo composition (Du et al., 2024). In contrast, pluripotent stem cell populations, including embryonic stem cells, maintain superior redox homeostasis and telomere stability, which is important to preserve EV biogenesis integrity and cargo quality (Saretzki et al., 2008; Dan et al., 2014; Barandalla et al., 2016).

Unlike embryonic or trophoblast stem cells, which are shielded from environmental exposures, adult stem cells are continually subjected to exogenous stressors. Environmental toxicants, pollutants, and dietary factors can amplify reactive oxygen species production, activate heat-shock and unfolded-protein responses, and impair stem-cell self-renewal (Hodjat et al., 2015; Eckhardt et al., 2022). In MSCs, this results in EVs enriched in stress-associated proteins and microRNAs with reduced regenerative potency (Huang et al., 2019; Li et al., 2020). Lipophilic pollutants can additionally incorporate into EV lipid bilayers, destabilizing membranes, impairing release, and altering uptake (Javdani-Mallak and Salahshoori, 2024). These exposures are far more relevant to adult stem cell-derived EVs, which reflect the cumulative impact of donor environment and aging, than to ESC- or TSC-derived EVs, which are relatively shielded from lifetime environmental burden (Hodjat et al., 2015). Taken together, these findings emphasize that controlling oxidative stress - both intrinsic to the stem cell and extrinsically from environmental exposures - is essential to preserve the regenerative and anti-aging properties of therapeutic EVs and highlight why adult stem cell-derived EVs show greater variability in quality than those from more developmentally primitive sources.

## 5 Clinical trials on stem cell-derived extracellular vesicles

Stem cell-derived EVs have begun to transition from preclinical promise to clinical investigation, with a growing number of early-phase trials exploring their safety and therapeutic potential across diverse medical fields (Wiklander et al., 2019). Clinical testing of EV-based therapeutics has been reviewed extensively elsewhere (Tan et al., 2024). One prominent example is the use of small EVs derived from human placental mesenchymal stromal cells (hPMSC-sEVs) in patients with COVID-19-associated acute respiratory distress syndrome (ARDS). In a double-blind, randomised, controlled trial, Zamanian et al. reported a substantial reduction in mortality among patients treated with hPMSC-sEVs (19%) compared to controls (54.2%), suggesting a potent immunomodulatory effect that may mitigate the severe inflammatory cascade of ARDS (Zamanian et al., 2024). The trial enrolled a total of 45 participants, allocating twenty-one to the treatment arm and twenty-four to the control arm, and demonstrated an acceptable safety profile across all three dose levels tested (Zamanian et al., 2024).

Beyond acute disease, regenerative and aesthetic applications have also drawn attention. A 3 years clinical trial led by Svolacchia et al. (2024) investigated EVs harvested from adult adipose-derived stem cells (Svolacchia et al., 2024). Their application resulted in visible improvements in dermal wrinkles and tissue firmness, achieved without eliciting an inflammatory response, which underscores the potential of EVs as a cell-free approach to tissue repair and cosmetic enhancement (Svolacchia et al., 2024). Further supporting the utility of EVs in aesthetic and dermatological indications, it has been reported that a 12-week regimen combining microneedling with a solution containing MSC-EVs from human adipose tissue led to significant reductions in facial aging markers, again with no reported safety concerns (Park et al., 2023). Additional evidence supports the use of EVs in cutaneous repair; a randomised double-blind controlled trial in 110 patients with chronic diabetic foot ulcers tested a Wharton’s jelly MSC-derived EV gel in addition to standard care against standard care with or without placebo vehicle. The EV-treated group showed greater reduction in ulcer area, a higher proportion of completely healed ulcers, with 62% of all participants achieving full re-epithelialization by the end of follow-up, and a markedly shorter median time to complete closure (6 vs. 20 weeks in controls), without any EV-attributable safety signal (Kishta et al., 2025).

Osteoarthritis, one of the most prevalent degenerative diseases of aging, has also begun to move into early clinical evaluation. A registered phase I trial in knee osteoarthritis is assessing the safety and exploratory efficacy of intra-articular injections of MSC-derived EVs (NCT05060107) (ClinicalTrials.gova, 2021). Complementing this, a randomised, double-blind, ascending-dose study reported that intra-articular administration of umbilical cord MSC-EVs was well tolerated and produced early signals of symptomatic improvement in pain and joint function, lending preliminary support to the regenerative potential of EV-based therapeutics in a condition that remains a leading cause of disability in older adults (Wang et al., 2025).

Neurological conditions are also beginning to feature in early clinical testing. The outcomes of a Phase 1 trial involving intrathecal administration of EVs derived from allogeneic human umbilical cord mesenchymal stem cells (HUC-MSCs) in patients with subacute complete spinal cord injury has been reported (Akhlaghpasand et al., 2024). Over a 12-month follow-up, improvements in sensory, functional, and bowel scores were observed in the cohort of 9 patients, and no treatment-related adverse events occurred, supporting the feasibility of intrathecal HUC-MSC-EV therapy in spinal cord injury (Akhlaghpasand et al., 2024). In acute ischaemic stroke, a major age-related neurovascular disorder, a Phase I/II clinical trial is currently registered to evaluate allogeneic MSC-EVs administered after infarction (NCT03384433), further signalling the extension of EV-based therapeutics into central nervous system diseases, although outcome data are not yet available (ClinicalTrials.govb, 2025).

Together, these studies provide encouraging early signals that stem cell-derived EVs may offer safe and effective interventions in contexts ranging from systemic inflammation and neurological injury to skin aging and complex wound healing. However, while the safety profile appears favourable across these trials, most remain small in scale and exploratory in nature. Robust, placebo-controlled studies with larger cohorts and longer follow-up will be essential to establish efficacy, define optimal dosing strategies, and guide regulatory pathways for clinical adoption.

## 6 Future perspectives

EVs isolated from stem cells have evolved as promising cell-free modalities for tissue engineering and regenerative medicine. Several factors affect the quality and functional capabilities of EVs, including the source of the cells, which is often overlooked. Moreover, oxidative stress, intrinsic changes in telomere shortening, and epigenetic changes can affect the quality of stem cells and, hence, the EVs isolated from them. Thus, there are significant changes in the immunological and functional properties of EVs depending on the stem cell source. The past several years witnessed tremendous progress in the development of advanced techniques for EV harvesting, engineering, and characterization, facilitating their potential use in targeted drug delivery applications and regenerative medicine. There are nearly 100 clinical trials that have been registered on clinicaltrials.gov, that are currently investigating the potential of EVs for various clinical applications. However, challenges remain that must be addressed before their clinical translation. Currently, there is a lack of standardized and reliable methods for EV isolation and characterization, which may negatively affect the consistency and reproducibility of EV-based therapeutics. From a clinical viability and commercialization perspective, EVs must be cost-effective to ensure they are accessible to the general population. Ideally, the source stem cells should be expandable using standard commercial media and the process should support large-scale production, efficient EV isolation, and thorough characterization. Another challenge is the variability in the quality of stem cell-derived EVs due to donor differences and challenges during the isolation procedures. This necessitates the development and implementation of automated, closed system workflows for EV isolation that are compliant with good manufacturing practice (GMP) standards and regulatory standardization during scale-up. A deeper mechanistic insight into EV function is essential for their successful transition from bench to bedside.
